# Enhancing vector refractoriness to trypanosome infection: achievements, challenges and perspectives

**DOI:** 10.1186/s12866-018-1280-y

**Published:** 2018-11-23

**Authors:** Henry M Kariithi, Irene K Meki, Daniela I Schneider, Linda De Vooght, Fathiya M Khamis, Anne Geiger, Guler Demirbaş-Uzel, Just M Vlak, ikbal Agah iNCE, Sorge Kelm, Flobert Njiokou, Florence N Wamwiri, Imna I Malele, Brian L Weiss, Adly M M Abd-Alla

**Affiliations:** 1 Biotechnology Research Institute, Kenya Agricultural & Livestock Research Organization, P.O Box 57811, 00200, Kaptagat Rd, Loresho, Nairobi, Kenya; 2 Molecular Department, Vector and Vector Borne Diseases Institute, Tanzania Veterinary Laboratory Agency, Majani Mapana, Off Korogwe Road, Box, 1026 Tanga, Tanzania; 3 Insect Pest Control Laboratory, FAO/IAEA Agriculture & Biotechnology Laboratory, IAEA Laboratories Seibersdorf, A-2444 Seibersdorf, Austria; 4 Laboratory of Virology, Wageningen University and Research, Wageningen, 6708 PB The Netherlands; 5 Department of Epidemiology of Microbial Diseases, Yale School of Public Health, 60 College Street, New Haven, CT 06510 USA; 6 Department of Biomedical Sciences, Institute of Tropical Medicine, Antwerp, Belgium; 7 International Centre of Insect Physiology and Ecology, P.O. Box 30772, 00100, Nairobi, Kenya; 8 INTERTRYP, Institut de Recherche pour le Développement, University of Montpellier, Montpellier, France; 9 Institute of Chemical, Environmental & Biological Engineering, Research Area Biochemical Technology, Vienna University of Technology, Gumpendorfer Straße 1a, 1060 Vienna, Austria; 10 Department of Medical Microbiology, Acıbadem Mehmet Ali Aydınlar University, School of Medicine, 34752, Ataşehir, Istanbul, Turkey; 11 Centre for Biomolecular Interactions Bremen, Faculty for Biology & Chemistry, Universität Bremen, Bibliothekstraße 1, 28359 Bremen, Germany; 12 Laboratory of Parasitology and Ecology, Faculty of Sciences, Department of Animal Biology and Physiology, University of Yaoundé 1, Yaoundé, BP 812 Cameroon; 13 Trypanosomiasis Research Centre, Kenya Agricultural & Livestock Research Organization, P.O. Box 362-00902, Kikuyu, Kenya

**Keywords:** *Glossina*, Microbiota, Paratransgenesis, Vector competence, *Trypanosoma*-refractoriness, sterile insect technique, *Hytrosaviridae*

## Abstract

With the absence of effective prophylactic vaccines and drugs against African trypanosomosis, control of this group of zoonotic neglected tropical diseases depends the control of the tsetse fly vector. When applied in an area-wide insect pest management approach, the sterile insect technique (SIT) is effective in eliminating single tsetse species from isolated populations. The need to enhance the effectiveness of SIT led to the concept of investigating tsetse-trypanosome interactions by a consortium of researchers in a five-year (2013–2018) Coordinated Research Project (CRP) organized by the Joint Division of FAO/IAEA. The goal of this CRP was to elucidate tsetse-symbiome-pathogen molecular interactions to improve SIT and SIT-compatible interventions for trypanosomoses control by enhancing vector refractoriness. This would allow extension of SIT into areas with potential disease transmission. This paper highlights the CRP’s major achievements and discusses the science-based perspectives for successful mitigation or eradication of African trypanosomosis.

## Background

Tsetse flies (Diptera; Glossinidae) transmit African trypanosomes across sub-Saharan Africa. These protozoan parasites are the causative agents of human and animal African trypanosomoses (HAT and AAT, respectively), which are neglected tropical diseases that are fatal if left untreated [1, 2]. A lack of effective prophylactic vaccines and drugs that target trypanosomes [3, 4] makes control of the tsetse vector an appealing alternative to reduce disease transmission. One attractive vector control method is the sterile insect technique (SIT), which is effective when included as a component of an area-wide integrated pest management (AW-IPM) approach [5–8]. SIT involves the mass production of sterilized male adults, which subsequently out-compete wild males in mating with wild virgin females in the field [9]. These matings are non-productive, eventually resulting in the decline and elimination of the target wild insect populations [10].

The successful and sustained eradication of *Glossina austeni* Newstead and AAT from Unguja Island in 1997 [7], in which SIT played a pivotal role, inspired African Governments to implement similar campaigns against tsetse on mainland Africa. SIT has also been employed to suppress *G. palpalis gambiensis* and *G. tachinoides* populations in Burkina Faso, *G. p. palpalis* in Nigeria [11, 12], and *G. pallidipes* in Ethiopia [13]. Challenges associated with improving SIT effectiveness include successful colony establishment [14], management of pathogenic infections that reduce colony fitness [15, 16] and compromised performance of field-released sterile males [17]. Importantly, the ability of released sterile males to vector trypanosomes increases the risk of transmitting disease in foci where trypanosomes are actively circulating. Furthermore, irradiation used for sterilization may negatively impact tsetse fitness (e.g. by damaging the tsetse host and its associated beneficial microbiota [18, 19].

## The joint FAO/IAEA-sponsored coordinated research projects

To enhance the SIT programs, the Joint Division of FAO/IAEA initiated a five-year (2013–2018) Coordinated Research Project (CRP) on enhancing tsetse fly refractoriness to trypanosome infections [20]. Composed of 22 research teams from 18 countries, the CRP involved four Research Coordination Meetings (RCMs) to review the results, progress and plan future research activities.

This paper highlights the major achievements towards answering the following four key research questions of the CRP: (1) Can the elucidation of tsetse-trypanosome molecular interactions assist in the development of novel methods and approaches to reduce or prevent the transmission of trypanosomes by irradiated tsetse flies? (2) Are tsetse’s symbiome and the fly’s competence as a vector of trypanosomes affected by radiation? (3) Can tsetse symbionts be used to develop novel vector and disease control tools, complementary to the SIT? (4) Can the characterization of tsetse’s symbiome and viral pathogens improve the efficacy of SIT? [20]. Many other concepts that emerged while addressing the above-mentioned research questions were addressed during the course of the CRP.

## Major objective of the CRP

The overall objective of the CRP was to elucidate the tsetse-symbiome-pathogen molecular interactions to improve SIT and SIT-compatible interventions. This effort was undertaken to reduce trypanosomosis by enhancing vector refractoriness, thus facilitating the expansion of SIT to areas where HAT-causing parasites are currently circulating in resident animals. The specific objectives and the expected output of this CRP are listed in Table 1 [See also Ref.#20]. The improved knowledge gained from achieving the objectives of the CRP is of significant interest to the FAO/IAEA and sub-Sahara African countries in their endeavor to control and ultimately eradicate tsetse and African trypanosomosis.

**Table 1:** The Five-year (2013–2018) CRP objectives, outputs and achievements (published papers)

Specific objectives	Expected output	Published papers^a^
(i). Elucidate tsetse-trypanosome interactions and understand determinants of vector competence.	(i). Molecular interplay of tsetse-trypanosomes characterized. (ii). Factors affecting trypanosome infections in tsetse determined. (iii). Tsetse vectorial competence assessed via comparative genomics and transcriptomics.	[102–146]; ([21, 26, 43, 107, 147])
(ii). Acquire better understanding of the physiology of tsetse-microbiota-pathogen tripartite interactions.	(i). Microbiota of multiple trypanosome-infected and uninfected tsetse species and hybrids determined. (ii). Trypanosome-microbiota interactions in model tsetse species and hybrids determined. (iii). Impacts of viral pathology on the tsetse symbionts determined.	[42, 47, 53, 58, 59, 148–172]; ([44, 54, 72, 93, 173, 174])
(iii). Determine effects of radiation in tsetse, its microbiota and pathogens.	(i). Effects of radiation on tsetse vectors, their symbionts and pathogens determined. (ii). Mutagenic effect of radiation on paratransgenesis determined.	[175]; ([95, 99])
(iv). Analyse SGHV-microbiota interactions in multiple tsetse species.	(i). Functional SGHV genes identified as candidates for developing antiviral mitigation strategy. (ii). Latency SGHV genes identified as tools for host interacting proteins. (iii). Mechanisms of SGHV’s escape from host defense response determined. (iv). SGHV haplotypes and evolution in lab-reared and wild tsetse fly populations determined.	[75, 76, 79, 81, 82, 84, 176, 177]; ([28, 77, 78, 80, 83])
(v). Develop novel symbiont-based, SIT-compatible anti-trypanosomiasis strategies.	(i). *Wolbachia*-based population suppression and/or replacement strategies assessed. (ii). Trypanosome-refractory paratransgenic tsetse lines developed.	[94, 108, 178]; ([109])

^a^ *Articles in round brackets are published in the current issue of the BMC Special Issue. The remaining articles in this table have either been or are submitted for publication elsewhere during the five years (2013–2018) CRP period*

## Current status and achievements

During the course of the CRP (2013–2018) more than seventy scientific papers, detailing experimentally derived data related to achieving the project’s objectives, were published in peer reviewed journals. This special issue includes several of these papers, findings from which are briefly summarized in this introductory chapter along with the overall outcome of the project and future perspectives.

### Tsetse species resolution

Correct taxonomic identification of insects is imperative for many reasons including the fact that studies conducted on different taxa may be reported by the same species (names), thus creating confusion. It is therefore important to properly identify field-captured tsetse species during characterization of their inhabiting microbial communities (including parasites, pathogens and symbionts). During the CRP, Augustinos and colleagues [21; this issue] evaluated the use of different molecular tools that can be used to efficiently and accurately distinguish distinct *Glossina* species using samples deriving from laboratory colonies and museum collections as well as all those collected in the field. The combined use of relatively inexpensive molecular genetic techniques, along with the identification of species specific microsatellites and mitochondrial and nuclear markers, will facilitate accurate identification of several tsetse species in the future.

### Tsetse-microbiota-trypanosome interactions and determinants of vectorial competence

**Fig. 1:**
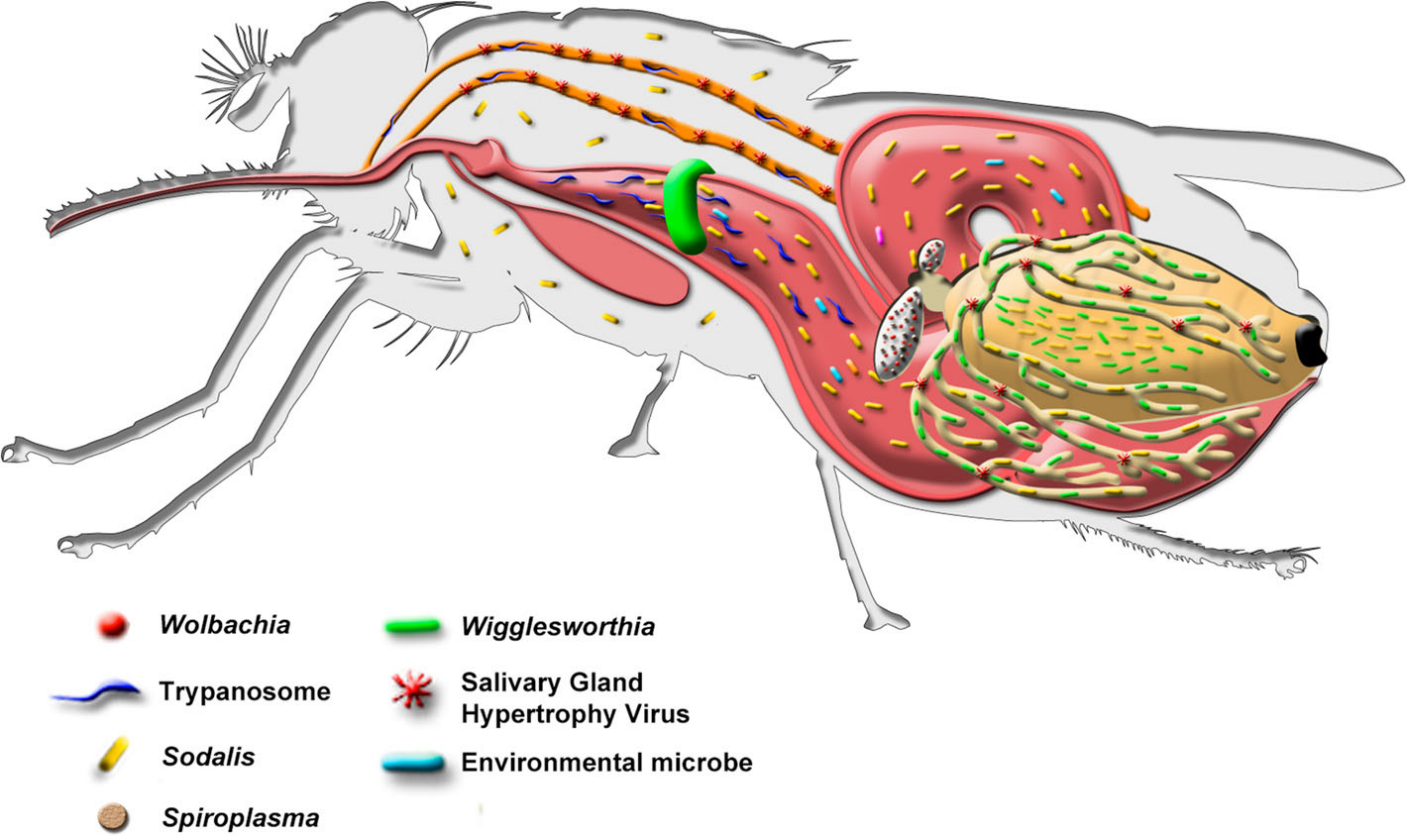
The tsetse fly and its associated microorganisms. Tsetse flies can harbor multiple microbes, including the bacterial endosymbionts obligate *Wigglesworthia*, facultative *Sodalis*, parasitic *Wolbachia* and *Spiroplasma*, as well as a taxonomically diverse population of environmentally acquired enteric bacteria, a virus (salivary gland hypertrophy virus, SGHV) and protozoan African trypanosomes. All tsetse harbor Wigglesworthia, while the presence of *Sodalis*, *Wolbachia*, *Spiroplasma*, SGHV and trypanosomes is fly population dependent. *Wigglesworthia*, *Sodalis* and SGHV are transmitted to developing intrauterine larval offspring via maternal milk secretions, while *Wolbachia* is transmitted through the germline. *Spiroplasma’s* mode of vertical transmission is currently unknown. Pathogenic trypanosomes are acquired by tsetse when they feed on an infected animal. The parasites must then undergo a complex development cycle in the fly before they can be successfully transmitted to a new host, where they cause disease. (This figure is adapted with permission from Aksoy et al., 2013) [179]

Figure 1 summarizes the interwoven associations and localization of the tsetse’s microbiota, which is comprised of the *Wigglesworthia*-*Sodalis*-*Wolbachia* complex, recently discovered *Spiroplasma*, environmentally acquired enteric bacteria, the salivary gland hypertrophy virus (SGHV) and the *Trypanosoma* parasite.

#### Trypanosome co-infections in tsetse flies

Molecular epidemiological surveys indicate that tsetse fly midguts, sampled from various HAT and AAT foci (including Fontem [22, 23] Campo and Bipindi [22, 24], Bafia [25] and Faro and Deo [26; this issue] in Cameroon) are infected with multiple trypanosome species. Application of nuclear ribosomal internal transcribed spacer (ITS) and/or trypanosome species-specific primers revealed that 53–82% of flies housed infections with trypanosome of a single species (*T. brucei* sl., *T. congolense* “forest” and “savannah” types, *T. vivax* and *T. simiae*), 18–47% were infected with two or three of the aforementioned species. In the Malanga HAT focus in Democratic Republic of Congo, 13.87% and 1.9% of *G. p. palpalis* had single and mixed trypanosome infections, respectively [27]. To assess the prevalence of trypanosome infection in a geographically broader area, Ouedraogo et al. [28; this issue] screened 3102 individual tsetse flies comprised of four species collected in five countries in west Africa. Results from this study indicate that trypanosome infections prevalence varied between tsetse species and location, but was on average substantial. In other words, infection prevalence ranged widely from 2.2–61.1% in flies sampled from different species in different locations. Furthermore, mixed infection was rarely observed (< 10%), and could be attributed to host specificity and/or preferences (human, domestic and wild animals) of particular tsetse species [29–32] and/or sensitivity of the PCR assay.

#### Modulations of tsetse gene expression during trypanosome infections

During SG infections, *T. b. brucei* suppresses the expression of the most abundant proteins in *G. m. morsitans* SGs, especially the proteins involved in the blood feeding process (e.g. Tsal1/2, TAg5, TSGF-1/2, 5’-Nuc, ADA and Spg3) [33]. This reduction in protein expression may significantly reduce fly feeding performance, consequently promoting vector competence via increase of the fly’s biting frequency. Further, the parasite upregulates expression of specific host proteins that are essential for parasite maturation, particularly proteins (e.g. CaMK, Serp-2, V-ATPases, and ArgK) involved in the regulation of stage-specific parasite differentiation [33, 34]. In response to the SG infection, tsetse overexpresses at least 15 immunity-related proteins [See Table 3 in Ref.#33]. In the midguts of *G. pallidipes*, which is more refractory to midgut colonization by trypanosomes compared to *G. m. morsitans* [35], *T. b. brucei*-challenge did not significantly modulate most of the genes (> 93%) in infected flies compared to uninfected controls [36]. However, whereas *T. b. brucei* induced expression of metabolism-associated genes in teneral flies (24 h post challenge), immunity-related and oxidative stress (ROS) genes were induced during late infection stages (48 h post challenge) [36]. Induction of expression of immunity and ROS genes is partially implicated in trypanosome-refractoriness in *G. m. morsitans* [37]. Notably, unlike in *G. m. morsitans*, in which only a small proportion of midgut infections progress to the SG, all *G. pallidipes* with trypanosome gut infections end up hosting mature SG infections [35]. Together, these data are applicable in designing strategies to interfere with metacyclogenesis and transmission of the mammalian-infective metacyclic (MT) parasites in the SGs of *G. pallidipes*. The SG tissue bottleneck (in trypanosome transmission) represents a vulnerable and attractive intervention point to enhance natural tsetse refractoriness to trypanosomes or to reduce the vectorial competence of the sterile males used in SIT campaigns.

#### Role of Sodalis in the establishment of trypanosome infections in tsetse midguts

*Sodalis glossinidius*, tsetse’s facultative endosymbiont, may modulate the ability of trypanosomes to establish an infection in tsetse’s midgut. However, the mechanism(s) that underlies this association is poorly understood [38–40]. This CRP addressed this knowledge gap by further exploring the relationship between *Sodalis* and trypanosome infection in tsetse. Geiger et al. [41] observed a correlation between specific *Sodalis* genotypes and tsetse’s ability to establish trypanosomes infection.

Hamidou et al. [42] demonstrated that *Sodalis*-hosted prophages also mediate trypanosome infection establishment by affecting *Sodalis* densities. However, certain studies on field-caught tsetse did not indicate any strong associations between *Sodalis* densities and trypanosome infections [26; this issue, 43; this issue]. In addition, a correlation between trypanosome infection and *Sodalis* presence observed in Kenya [43; this issue] was weak or nonexistent. However, the authors thought that tsetse-trypanosome-microbiota interactions could be influenced by other factors such as tsetse’s ecology and community compositions, but only in some species of trypanosomes. However, Griffith et al., [44; this issue] found that *Sodalis* densities were significantly higher in trypanosome-infected, wild-caught flies compared to their uninfected counterparts. Additionally, other confounding factors may indirectly affect vectorial competence, including tsetse flies age, sex, habitat, species of trypanosome, and *Sodalis* genotypes and their modulation of the host’s immune system [43, this issue]. These factors may influence *Sodalis* densities, which may indirectly impact trypanosome prevalence within tsetse and the fly’s vectorial competence for trypanosome transmission.

##### Insights into tsetse-microbiota-pathogen tripartite interactions

ᅟ

### Tsetse symbionts

#### Taxonomic composition of microbial communities housed in the gut of wild tsetse

Enteric microbes impact several aspects of their host’s physiology [45]. In tsetse, the obligate mutualist *Wigglesworthia* mediates numerous aspects of the fly’s physiology, including nutrition, reproduction and immune system maturation and function [46–48]. Over the course of this CRP, researchers performed studies to characterize the taxonomic composition of environmentally acquired bacteria housed in the gut of field-captured and colonized tsetse. This information is an important prelude to understanding how this population of microbes impacts tsetse’s fitness and susceptibility to trypanosome infection. Using culture dependent and independent techniques, prominent bacterial taxa found in guts from field captured tsetse included *Serratia*, *Enterobacter*, *Enterococcus*, *Acinetobacter*, *Providencia*, *Sphingobacterium*, *Chryseobacterium*, *Lactococcus*, *Staphylococcus*, and *Pseudomonas*, *Bacillus*, *Mesorhizobium, Paracoccus, Microbacterium, Micrococcus, Arthrobacter, Corynobacterium, Curtobacterium, Vagococcus,* and *Dietzia* spp. ([44, 49–54]; this issue). The sources and mechanisms by which tsetse flies acquire this diverse enteric microbiota remain unclear. However, tsetse hosts from specific ecosystems could differ in their microbial diversities [55]. Flies could ingest bacteria present on host skin when probing for a blood meal [56], or host blood may contain bacteria that are ingested by flies during feeding on a septic host. Identification of diverse bacteria in tsetse tissues that also house trypanosomes raises the question whether these bacteria influence trypanosome infections. Environmentally acquired bacteria found in the gut of other disease vectors (i.e., *Anopheles gambiae*) exhibit direct anti-parasitic properties [57]. As such, tsetse’s gut microbiota should be explored in more detail to determine if bacteria that exhibit anti-trypanosomal properties are present in the fly’s gut.

#### Discovery and characterization of Spiroplasma, a potential fourth symbiont of tsetse

One of the major achievements of this CRP is the discovery of *Spiroplasma* as a fourth endosymbiont (in addition to *Wigglesworthia*, *Sodalis*, and *Wolbachia*) in some wild and laboratory-reared tsetse populations [58, 59]. While the function of this bacterium in tsetse is currently unknown, it likely to impact colony fitness. However, in *Drosophila*, *Spiroplasma* is a maternally [60] and horizontally transmitted mutualist [61]. Some lineages of *Spiroplasma* confer their hosts with important traits, including defense against pathogens (e.g. parasites and bacteria), either singly or in associations with other symbionts such as *Wolbachia* [62–65]. The poorly understood mechanism(s) of *Spiroplasma-Wolbachia* associations presents an intriguing research topic, given that *Wolbachia* (found mainly in reproductive organs) and *Spiroplasma* (resides primarily in the hemolymph, but can also invade other tissues such as ovaries, fat body and SGs) exhibit similar tissue tropisms. Research on the *Glossina*-*Spiroplasma* association is required to determine if the bacterium presents commensal, mutualist or pathogenic phenotypes in the fly. Additionally, it will be important to determine the relationship between *Spiroplasma* and other constituents of tsetse’s microbiota, including bacterial symbionts, viral pathogens and trypanosomes. Finally, studies should be performed to determine if *Spiroplasma* can be utilized to develop novel symbiont-based strategies aimed at blocking trypanosome transmission.

#### Role of Wolbachia in tsetse speciation and generation of fertile hybrid tsetse colonies

Symbiont-induced cytoplasmic incompatibility (CI) acts as an efficient post-mating barrier to hybrid formation, making it an important parameter in preserving species borders [66–69]. In tsetse, *Wolbachia* efficiently triggers CI within [70] and between species [71]. During the CRP, *Wolbachia* related research focused on two main topics: 1) the development of diagnostic tools sensitive to detect low titer *Wolbachia* infections in tsetse species, and 2) exploration of *Wolbachia’s* role in tsetse speciation. In relation to the first topic, Schneider et al. [72; this issue], compared classic endpoint PCR with high-sensitivity blot-PCR and demonstrated that the latter technique facilitates more sensitive detection of low-titer *Wolbachia* in the *morsitans* and *palpalis* groups than does classic endpoint PCR. In addition, the authors used a high-end Stellaris® rRNA-FISH based technique to localize *Wolbachia* in situ in high and low-titer *Glossina* species, and demonstrated that with this highly sensitive method, even low amounts of *Wolbachia* can be traced in specific tissues. The results also highlight that more tissues and organs than previously recorded are infested with *Wolbachia* in subspecies of the *morsitans* and *palpalis* groups. The novel, highly sensitive molecular *Wolbachia* detection tools developed during the CRP [72; this issue] should expedite further investigations on the tsetse hybrid colonies.

With regard to *Wolbachia’s* role in tsetse speciation, previously published data indicate that mating between *Wolbachia*-free *G. morsitans* females and wild type *G. morsitans* males results in significantly reduced larval deposition and adult eclosion rates [70]. Similarly, mating between wild type *G. morsitans* and *G. centralis* triggers high CI levels due to the presence of two incompatible *Wolbachia* strains [71]. However, premating barriers to hybrid formation are rather weak or completely absent, as members of various *Glossina* species mate readily [73]. Nevertheless, the negative effects of CI led to the consideration of generating tsetse hybrids for population control [74]. This consideration is based on the assumption that among artificially created hybrids between closely related *Glossina* species, males are post-zygotically incompatible with both parental species due to their natural hybrid sterility. Such pseudo-sterile tsetse males can be complementary to the SIT programs. Experiments performed during the CRP demonstrated that knockdown of native *Wolbachia* in *G. m. morsitans* males prior to their mating with *G. m. centralis* females results in successful establishment of a hybrid line, which is now maintained in the IPCL tsetse production facility in Seibersdorf, Austria (unpublished data). Therefore, prior to employing hybrid flies to existing SIT programs, further investigations are necessary to determine how symbiont status and mating competence are affected in the hybrid background, and whether the hybrids and wild type flies are equally fit.

#### Tsetse fly pathogens

In addition to microbial communities associated with tsetse flies, pathogens such as the SGHV (*Hytrosaviridae*) and entomopathogenic fungi (EPF) infect tsetse flies and hence affect fly fitness both in insect mass rearing facilities and in the field. During the CRP, research was conducted to gain a better understanding on the impact of these pathogens on tsetse fly fitness and susceptibility to trypanosomes.

#### Salivary gland hypertrophy viruses

### Pathobiology of GpSGHV haplotypes and the prospects for integrated antiviral strategies

Over the course of the CRP, the following topics related to SGHV were investigated: 1) improvement of virus control strategies [75], 2) explore genomic differences between virus isolates [76], 3) virus host range [77], 4) the impact of virus infection on tsetse fitness, 5) genetic diversity of field collected viral isolates, and 6) the impact of virus infection on the expression of tsetse immune genes. Comparative analyses of the Ethiopian and Ugandan GpSGHV strains [76] suggest that the differential virus-pathologies (i.e. outbreaks of the salivary gland hypertrophy symptoms, SGH) in *G. pallidipes* colonies are due to factors such as differences in viral gene contents, host genetics and ecologies, and virus-host co-evolutionary histories [78]; this issue. GpSGHV pathological effects and the host’s response to the virus infection vary amongst different *Glossina* species. For instance, in *G. pallidipes*, GpSGHV infection results in significant upregulation of host genes associated with pathways promoting viral infection compared to upregulation of genes associated with antiviral responses in virus-infected *G. m. morsitans* [79]. We now have clues that more GpSGHV strains exist in multiple *Glossina* species, and that *G. pallidipes* may influence GpSGHV evolution [78, 80]; this issue. Susceptibilities of tsetse to GpSGHV infections, and the negative impacts of viral infections on the fly’s fecundity, adult eclosion and survival, differ amongst different fly species [77, 81]; this issue. The narrow GpSGHV host range (only in *Glossina* species) and lack of overt SGH in the majority of tsetse hosts do not preclude implementing precautionary antiviral measures in tsetse production facilities that rear multiple species [15, 16, 78, 82].

### Insights into the roles of tsetse immunity during symptomatic GpSGHV infections in lab-bred tsetse colonies

We have ascertained that GpSGHV infection provokes the RNA interference (RNAi) defense response, as evidenced by significant upregulation of the expression of key RNAi pathway genes (*Ago-1*, *Ago-2* and *Dcr-2*) in virus-injected flies (asymptomatically infected) compared to the non-infected flies [83; this issue]. These data imply that both siRNA and miRNA pathways (two of the RNAi machinery pathways) provide antiviral defense in asymptomatic infected flies, but the pathways are highly compromised during symptomatic infections. The third RNAi machinery pathway (piRNA pathway) appeared not to be involved in tsetse’s defense mechanism against GpSGHV, as virus infection did not affect the expression of *Ago-3* gene, a key gene in the piRNA pathway [83]. In addition to the RNAi, we have indications that GpSGHV infection alters the host miRNA profile in *G. pallidipes*, thus indicating possible functional importance of miRNAs in symptomatic infections [84; MS in Prep.]. Notably, the majority of the upregulated miRNAs were predicted to target over 700 host mRNAs, of which 150 mRNAs were immune-related. miRNA expression profiles are also modulated by the insect microbiota, and may therefore contribute to the outcomes of virus infection as has been demonstrated in the dengue mosquito vector *Aedes aegypti* [85]. Recent data suggest that the absence (or low densities) of *Wolbachia* positively correlates with SGHV outbreaks in *G. pallidipes* colonies compared with other *Glossina* species that rarely exhibit overt SGH symptoms [86]. Whether differences in *Wolbachia* prevalence in tsetse species is linked to differences in GpSGHV infections (e.g. via modulations of miRNAs) requires further investigations.

### Entomopathogenic fungi

EPF have been proposed as potential mosquito control agents [87]. The EPF *Metarhizium anisopliae* (Metsch.) Sorok may suppress wild tsetse populations when autodisseminated from devices mounted on pyramidal traps [88]. Furthermore, horizontal transmission of the EPF was demonstrated between *M. anisopliae*-infected *G. pallidipes* and fungus-free flies during mating [89]. These characteristics make *M. anisopliae* a suitable candidate to be combined with SIT. Prior to causing death, fungal infection can significantly reduce tsetse feeding and reproduction [90–92]. Therefore, the complementary action of EPF on reducing tsetse’s blood feeding and reproduction capacity, and potential effects on trypanosome development within the vector, could influence disease epidemiology and transmission. During the CRP, Wamiti et al. [93; this issue] conducted research focused on determining the impact of EPF on trypanosome infection. The results indicate that infection of *G. f. fuscipes* with *M. anisopliae* resulted not only in significant reduction in *T. congolense* titers, but also hindered the fly’s vectorial competence (ability to acquire and transmit trypanosomes to mice). The precise mechanism(s) underlying the fungal-mediated anti-trypanosome impacts remain to be elucidated.

### Effects of irradiation on tsetse, its microbiota and trypanosome infections

One of the major objectives of this CRP was to investigate the possibility of combining paratransgenesis with SIT to control tsetse population size and simultaneously reduce their vector competence. Paratransgenesis involves genetically modifying tsetse’s commensal endosymbiont *Sodalis* so that it produces anti-trypanosome factors. Modified *Sodalis* are reintroduced into female flies, which subsequently present a trypanosome refractory phenotype ([94]; see section “Prospects of developing symbiont-based anti-trypanosome strategies” below for more details). As sterile males are produced via exposure to irradiation, the impact of this treatment on modified *Sodalis* is crucial for the implementation of the combined approach. To this end, Demirbaş-Uzel et al. [95; this issue] investigated the correlation between tsetse developmental stage (22- day old pupae, 29-day old pupae and 7-day old adults) at the time of radiation exposure and impact on *Sodalis* density. The results indicate that irradiation of seven-days old *G. m. morsitans* adults significantly reduced *Sodalis* densities. Furthermore, the recovery of *Sodalis* densities was significantly higher in the adults that emerged from puparia that had been irradiated on day 22 post larviposition as compared to the flies that had been irradiated as adults [95]. Results also indicate that irradiation of puparia on day 22 post larviposition has no effect on the vectorial capacity of the emerged males to transmit trypanosomes. The recovery of *Sodalis* titers in sterile males opens the door to combine paratransgenesis with SIT for tsetse control. In addition, pupal irradiation is operationally advantageous in terms of handling and transportation compared to adult irradiation [96].

Field released sterile males must efficiently identify and mate with wild females. Therefore, one component of the CRP investigated the effects of various doses of ionizing radiation on tsetse cuticular hydrocarbon (CHCs; e.g. n-alkanes, alkenes and methyl-branched hydrocarbons) profiles. CHCs act as sex pheromones for species, sex, and mate recognition in *Drosophila* [97] and tsetse [98]. Engl et al. [99; this issue] investigated the impact of bacterial symbionts and irradiation on tsetse CHC profiles. They discovered that antibiotic-mediated knockdown of tsetse’s indigenous microbiota significantly reduced tsetse’s CHCs profiles and correspondingly impacted mate choice. [99; this issue]. However, no significant differences in CHC profiles were observed between irradiated and non-irradiated *G. m. morsitans* flies [99]. These findings call for further research into the roles of microbiota (e.g. *Wigglesworthia*) in tsetse’s mating behavior (in terms of CHC synthesis), and how the effects of irradiation on the microbiota can be reversed in irradiated males before inundative releases during SIT applications.

### Prospects of developing symbiont-based anti-trypanosome strategies

**Fig. 2:**
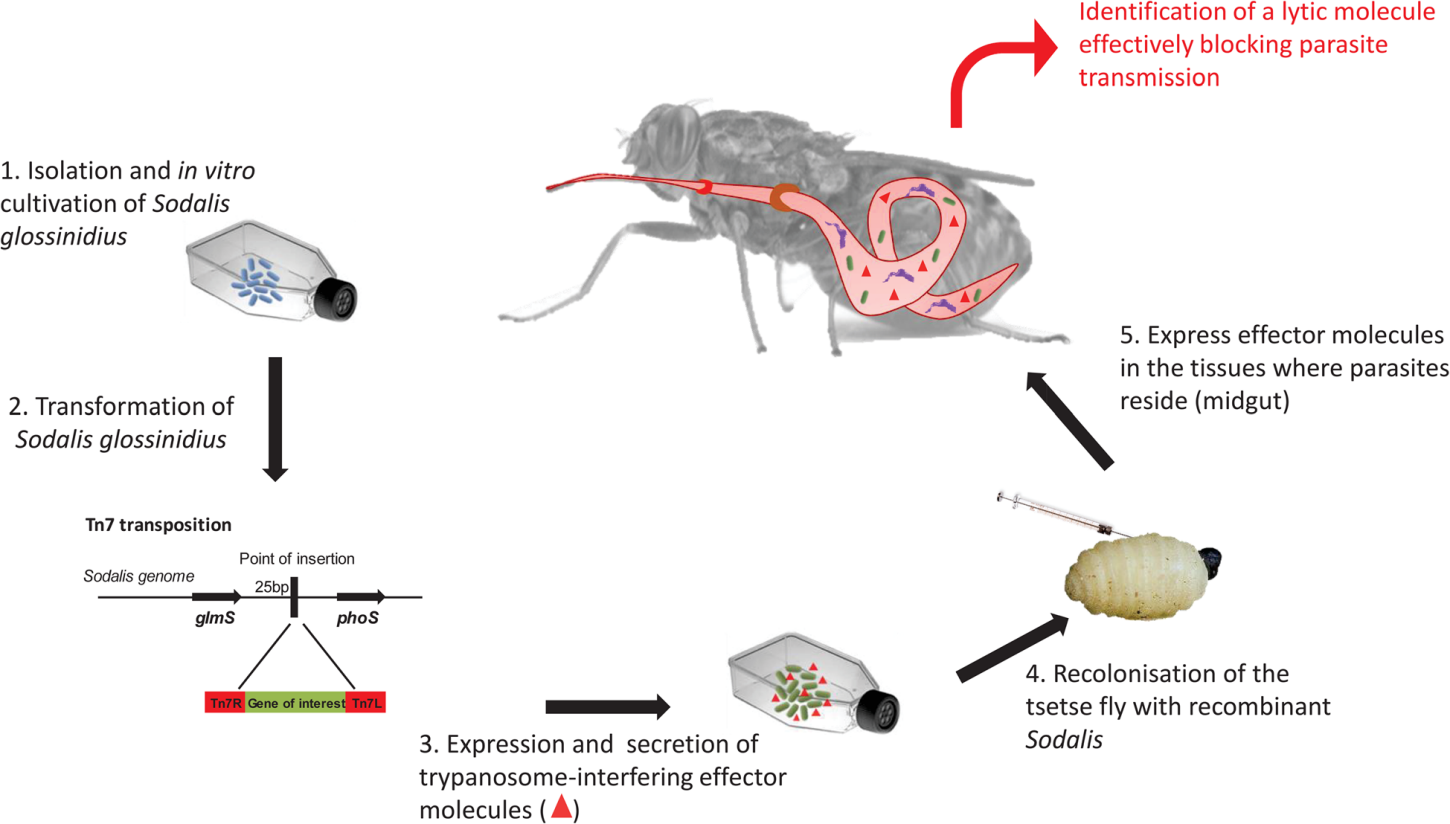
Overview of the current status on tsetse paratransgenesis. Strategies have been developed for i) isolation and *in vitro* cultivation of *Sodalis glossinidius*, ii) establishing stable chromosomal expression in *Sodalis* allowing strong and constitutive expression of anti-trypanosome compounds in the absence of antibiotic selection and iii) the sustainable colonization of tsetse fly and its subsequent generations with genetically modified *Sodalis* through microinjection of the bacterium into third-instar larvae [109; this issue]. Taken together, the necessary technology for application of *Sodalis* as a delivery system in tsetse paratransgenic has been developed, but the *Sodalis*-mediated inhibition of parasite development in the insect host is yet to be demonstrated. The final main bottleneck remains the identification of a highly potent and stable trypanolytic component effectively blocking parasite transmission by the fly without impairing symbiont and vector fitness

The development of trypanosome-refractory sterile males would make SIT much less controversial, particularly when applied in trypanosome-endemic locations [20]. The viviparous reproduction of tsetse is not directly amenable to germ-line transformation for the purpose of ectopically expressing trypanocidal transgenes in an effort to reduce the fly’s vector competence [100]. However, trypanosome-refractoriness can be indirectly conferred to tsetse via paratransgenesis, whereby genetically engineered symbionts express molecules that block trypanosome development and/or transmission [101] **(**Fig. 2**)**. This approach works in triatome bugs [102] and mosquitoes [103, 104]. *Sodalis* is an ideal bacterium for expressing effector molecules in paratransgenic tsetse because it (i) resides in close proximity to trypanosomes; (ii) can be cultured and engineered *in vitro*; (iii) can be re-introduced into tsetse after transformations; (iv) is maternally transmitted to fly progenies, and (v) is rigorously restricted to the tsetse host niche [105]. Engineered *Sodalis* can express and release significant amounts of functional nanobodies that target trypanosome surface epitopes in different tsetse tissues [94, 106]. Moreover, improved strategies have been developed to: (i) identify and determine population dynamics of tsetse species in a particular area [107; this issue], (ii) establish stable chromosomal expression in *Sodalis* allowing strong and constitutive expression of anti-trypanosome compounds [108], and (iii) sustainably colonize tsetse and its subsequent generations with genetically modified *Sodalis* through microinjection into third-instar larvae [109; this issue]. *Sodalis*-mediated inhibition of parasite development in paratransgenic tsetse remains to be demonstrated.

## Conclusions

A large body of information related to enhancing tsetse fly refractoriness to trypanosome infections was acquired over the course of this CRP. However, many challenges and questions remain, which include, but are not limited to 1) developing more efficient tools to correctly classify field captured tsetse flies, 2) further deciphering the functional association between tsetse’s microbiota (including environmentally acquired enteric bacteria, endosymbiotic microbes and pathogenic or symbiotic viruses and fungi) and the fly’s physiology and trypanosome vector competency, 3) optimizing SIT irradiation protocols so that the treatment has a minimal effect of tsetse/endosymbiont fitness, and 4) maximizing the efficiency of tsetse paratransgenesis. Theoretical and technical knowledge acquired from experiments performed using the model tsetse species, *G. m. morsitans* (and its associated microorganisms), serves as a foundation for similar studies in other, more epidemiologically relevant tsetse species.

This CRP served as a platform for scientists from African, European and North American countries to interact, exchange ideas and develop long-term, mutually beneficial collaborations. Additionally, the extensive collaborations established during the CRP will continue in a new five-year CRP, which will address various issues related to the improvement of colony management in tsetse mass rearing for SIT applications (http://www-naweb.iaea.org/nafa/ipc/crp/new-crps-ipc.html). Finally, African members of this CRP can disseminate knowledge and expertise acquired to additional research communities in other tsetse-endemic regions of sub-Saharan Africa and to national authorities to promote the novel insights in tsetse and trypanosomosis control.

## Abbreviations

16S rRNA: 16S ribosomal RNA
5’-Nuc: 5′-nucleotidase-related saliva protein
ADA: Adenine deaminase
Ago: Argonaute
AMP: Antimicrobial peptide
ArgK: Arginine kinase
AW-IPM: Area-wide integrated pest management
BSF: Bloodstream form
CaMK: Ca2+/calmodulin-dependent protein kinase
CHCs: Cuticular hydrocarbons
CI: Cytoplasmic incompatibility
CRP: Coordinated research project
Dcr: Dicer
DEG: Differentially expressed gene
DENV: Dengue virus
dsRNA: Double-stranded RNA
EPF: Entomopathogenic fungus
FAO: Food and Agricultural Organization of the United Nations
GpSGHV: *Glossina pallidipes* salivary gland hypertrophy virus
HGT: Horizontal gene transfer
IAEA: International Atomic Energy Agency
Imd: Immune deficiency
MdSGHV: *Musca domestica* salivary gland hypertrophy virus
miRNA: Micro RNA
MLST: Multi locus sequence typing
MT parasites: Mammalian-infective metacyclic parasites
PGRP-LB: Peptidoglycan-recognition protein LB
piRNA: Piwi-interacting RNA
RCM: Research Coordination Meetings
RNAi: RNA interference
RNA-Seq: Ribonucleic acid (RNA) sequencing
ROS: Reactive oxygen species
SG: Salivary gland
siRNA: Short interfering RNA
SIT: Sterile insect technique
Spg3: 5′-nucleotidase-related SG protein-3
TAg5: Salivary antigen-5-protein
Tbb: *Trypanosoma brucei brucei*
Tbg: *Trypanosoma brucei gambiense*
Tbr: *Trypanosoma brucei rhodesiense*
Tc: *Trypanosoma congolense*
Tsal1/2: Tsetse salivary gland proteins 1 & 2
TSGF-1/2: Tsetse salivary growth factors 1 & 2
V-ATPase: Vacuolar-type H^+^ -ATPase
